# Sn–Ag–Cu nanosolders: Melting behavior and phase diagram prediction in the Sn-rich corner of the ternary system

**DOI:** 10.1016/j.calphad.2015.04.003

**Published:** 2015-06

**Authors:** Ali Roshanghias, Jan Vrestal, Andriy Yakymovych, Klaus W. Richter, Herbert Ipser

**Affiliations:** a Department of Inorganic Chemistry (Materials Chemistry), University of Vienna, A-1090 Vienna, Austria; b Masaryk University, CEITEC MU, Brno, Czech Republic

**Keywords:** Nanoparticles, CALPHAD, Lead free solders, Size effect, Melting point depression

## Abstract

Melting temperatures of Sn–Ag–Cu (SAC) alloys in the Sn-rich corner are of interest for lead-free soldering. At the same time, nanoparticle solders with depressed melting temperatures close to the Sn–Pb eutectic temperature have received increasing attention. Recently, the phase stability of nanoparticles has been the subject of plenty of theoretical and empirical investigations. In the present study, SAC nanoparticles of various sizes have been synthesized via chemical reduction and the size dependent melting point depression of these particles has been specified experimentally. The liquidus projection in the Sn-rich corner of the ternary SAC system has also been calculated as a function of particle size, based on the CALPHAD-approach. The calculated melting temperatures were compared with those obtained experimentally and with values reported in the literature, which revealed good agreement. The model also predicts that with decreasing particle size, the eutectic composition shifts towards the Sn-rich corner.

## Highlights

• The first CALPHAD-type modeling of a ternary system that includes the size effect.
• Sn–3.8Ag–0.7Cu nanoparticles of different size were synthesized via chemical reduction.
• The liquidus projection in the Sn-rich corner was calculated as a function of particle size.
• The size dependent melting depression behavior of nanoparticles was verified.
• The eutectic composition changed towards the Sn-rich corner with decreasing particle size.

## 1 Introduction

Considerable research has been focused on the synthesis of pure Sn [1–3] as well as near-eutectic Sn–Ag (SA) [4,5] and Sn–Ag–Cu (SAC) [4,6–11] nanoparticles as promising candidates for the next generation of lead-free solders due to their reduced melting temperatures. The application of various techniques such as chemical and sonochemical reduction, ball milling and the arc discharge method in these studies lead to the fabrication of tin-based nanoparticles with different sizes and thereby different melting temperatures [3–11]. As a matter of fact, melting point depression in nanosolders was the main interest of most of these investigations, since the melting point of eutectic SAC bulk alloys is approximately 217 °C, about 34 °C higher than that of the conventional Sn–Pb solders [9].

Various models such as the Gibbs–Thomson, the homogeneous melting model (HMM) and the liquid skin melting model (LSM) [12,13] have been previously proposed to describe the size dependence of the melting temperature of nano-sized particles [11]. It is believed that, as the melting is initiated by a continuous vibrational lattice instability on the solid surface, nanoparticles with large surfaces will lose their stability at lower temperature than bulk. The melting point depression for nanoparticles can be described for instance in a classical thermodynamic approach by the Gibbs–Thomson equation [12], as follows:

(1) $$T_{m}^{Nano}\left(r\right)=T_{m}^{bulk}-\frac{2\left(T_{m}^{bulk}+273.15\right)\sigma_{sl}}{\Delta H_{f}^{bulk}\rho_{s}r}$$

where $\sigma_{sl}$ is the solid–liquid interfacial energy (∼820 J/cm^2^), $\rho_{s}$ is the solid phase density of bulk alloy (7.39 g/cm^3^) and, $\Delta H_{f}^{bulk}$ is the latent heat of fusion of the bulk alloy (∼67 J/g) for the eutectic SAC composition [14], *r* is the particle radius and *T* is melting point in °C. Other models predict an essentially similar relationship as the Gibbs–Thomson equation, where $T_{m}$ varies linearly with the reciprocal radius, however the surface tension term has been shown to differ slightly among the models [10–12].

The CALPHAD (Computer Calculation of Phase Diagrams)-type thermodynamic description of alloy nanoparticles is a powerful tool for predicting the phase diagram of nanoparticles equal to or larger than 5 nm in radius [15]. For the smaller particle size in the range of a few isolated atoms, a bottom-up calculation such as molecular dynamics can be adopted [16]. The CALPHAD-type thermodynamic modeling of a nano-sized alloy system was first introduced by Park and Lee [17]. However, the phase diagram reassessment of nanoparticles has been so far confined to fully miscible alloys or simple eutectic alloy systems such as the Ag–Au [17] and Cu–Ni [18] binary systems. For an incongruently melting system containing intermetallic compounds like the Sn–Ag system, Sim and Lee [15] performed recently a systematic study, in which the standard Gibbs free energy of the stoichiometric compound (Ag_3_Sn) was separately calculated based on the existing data for the surface tension of Ag_3_Sn.

The ternary SAC eutectic alloy solder has several advantages over the binary SA eutectic solder, due to its lower eutectic temperature (217.2 °C compared to 221.8 °C), slower growth of the intermetallic layer at the interface, increased strength, and a lower wetting angle [19]. Since the melting temperatures of the ternary SAC alloys in the Sn-rich corner are of interest for lead-free soldering, and numerous experimental studies on the phase stability of SAC nanoparticles have been performed, the CALPHAD-type thermodynamic modeling of the binary SA nano-system has been extended to the ternary SAC nano-system in this study. The present model is a modified version of the original model suggested by Park and Lee [17], employing latest updates on the Sn–Ag binary nanoalloy phase diagram [5,15] and including a reassessment of thermodynamic parameters for Sn–Cu and Ag–Cu binary nano-systems. In addition, Sn–3.8Ag–0.7Cu nanoparticles of different sizes have been synthesized via a chemical reduction technique by employing PVP as surfactant agent [20], thereby the theoretically predicted melting point temperature of nano-SAC alloys was verified.

## 2 Experimental procedure

Tin-based, near eutectic (Sn-3.8 wt% Ag-0.7 wt%Cu) nano-powder alloys have been synthesized via a chemical reduction technique by employing sodium borohydride (NaBH_4_; Alfa-Aesar) and polyvinylpyrrolidone (PVP; Alfa-Aesar) as reducing agent and surfactant agent, respectively. Correspondingly, stoichiometric amounts of tin (II) 2-ethylhexanoate (C_16_H_30_O_4_Sn; Alfa-Aesar), silver nitrate (AgNO_3_; Merck) and copper (II) nitrate trihydrate (Cu(NO_3_)_2_·3H_2_O; Merck) were dissolved in diethylene glycol (DEG; Alfa-Aesar) as the metal precursors [20]. PVP and NaBH_4_ were dissolved separately in DEG and rapidly added to solutions of the metal precursors and then stirred for 1 h. The molar ratio of NaBH_4_ to SAC powder was selected as (6: 1) for a typical 1 g metallic powder synthesis. Analogously, various molar ratios of PVP to SAC (*n_PVP_*/*n_SAC_*≤1) were added to the solution to control the morphology and size distribution of the powders. In this study, five different molar ratios of PVP (*n_PVP_*/*n_SAC_*=0, 0.05, 0.2, 0.35 and 0.4) have been selected for the synthesis of nanoparticles of different sizes. After the reaction was completed, the obtained precipitates were separated from the organic residue by centrifugation at 4000 r/min for 30 min, rinsed several times with a large amount of absolute ethanol to remove the excess amount of surfactant, filtered and finally dried in vacuum for one day at room temperature.

For particle size analysis, the nanoparticles were dispersed in ethanol, and a drop of this dispersion was trickled on a polished Si wafer for scanning electron microscopy (SEM) or on a copper grid coated with carbon film for transmission electron microscopy (TEM) investigations. SEM studies were performed on a scanning electron microscope (Zeiss Supra 55 ESEM). TEM investigations were made by utilizing a (FEI TECNAI F20 TEM) microscope equipped with energy-dispersive spectroscopy (EDS) and energy-loss spectroscopy (EELS). X-ray diffraction (XRD) patterns were obtained on a D8 Advance X-ray diffractometer (Bruker). For differential scanning calorimetry (DSC) a TG-DSC instrument (Labsys, SETARAM, Lyon) was used and experiments were carried out over the temperature range from room temperature to 650 °C at a heating rate of 5 K/min under the flow of Ar gas. The instrument was calibrated by comparison of the melting temperatures of metallic In, Sn and Cu (99.999 mass% purity) with tabulated values with an accuracy of the melting temperature of ±0.5 °C.

### 2.1 Phase diagram modeling

Nanoparticles have an increasing surface to volume ratio with decreasing particle size and therefore add a substantial contribution of the surface energy to the Gibbs energy in thermodynamic considerations.

The CALPHAD method is based on the minimization of the molar Gibbs energy of the entire system, which is a sum of the molar Gibbs energy of the phases present in the system. The total Gibbs free energy of each phase in a ternary system is given by [17]:

(2) $$G=G^{Bulk}+G^{Surface}$$

where $G^{Bulk}$ stands for the Gibbs energy of the bulk ternary alloy and $G^{Surface}$ denotes the surface excess Gibbs energy contribution.

### 2.2 Bulk phase diagram modeling

The general equation of the molar Gibbs energy for solution phases such as liquid (L) and disordered solid solutions in a ternary system is based on a substitutional solution model, expressed as follows [21,22]:

(3) $$G^{Bulk}=G^{ref}+G^{id}{+}^{ex}G^{binary}{+}^{ex}G^{ternary}$$

The first term $G^{ref}$ in Eq. (3) defines a surface of reference as:

(4) Gref=∑i=13xiGi0

where $x_{i}$ stands for the molar fraction of the element *i* (Sn, Ag, Cu) and Gi0 is the molar Gibbs free energy of the pure component. The second term in Eq. (3) is associated with the molar configurational entropy, which can be described as following:

(5) $$G^{id}=RT\sum_{i=1}^{3}x_{i}\;\ln\;x_{i}$$

where *R* is the gas constant and *T* is the absolute temperature. The third and fourth terms in Eq. (3) are the excess Gibbs energy contributions from binary $\left(G^{ex,binary}\right)$ and ternary interactions $\left(G^{ex,ternary}\right)$ expressed as Redlich–Kister-type equations:

(6) Gbinaryex=∑i=12∑j=i+13xixj(xi−xj)ϑLϑ,ij(ϑ=0,1,2,…)

(7) $$L_{\vartheta ,ij}=a+bT+cT\ln T+dT^{2}$$

(8) Gternaryex=xixjxk(xi.L0,ijk+xj.L1,ijk+xk.L2,ijk)

where $L_{\vartheta ,ij}$ are the binary interaction parameters from the binary systems and $L_{\theta ,ijk}\left(\theta =0,\;1,\;2\right)$ are the ternary interaction parameters which are all modeled in power series of *T*. Several thermodynamic studies of the bulk Sn–Ag–Cu ternary system have been published [22–24]. This study uses the parameters provided by Dinsdale et al. [24] which gave a ternary eutectic in the Sn-rich corner for the bulk alloy at 217.7 °C and Sn-3.29 wt% Ag-0.85 wt% Cu.

### 2.3 Nanoparticles phase diagram modeling

When the nanoparticle is assumed to be an isotropic spherical particle, the Gibbs energy of the surface $G^{Surface}\;$is expressed by [17]:

(9) $$G^{Surface}=\frac{2\sigma V}{r}$$

where V is the molar volume, r is the radius of the particles, and σ is the surface tension.

The general size-dependent Gibbs free energy description of all the phases and their modified interaction parameters for the nano-SAC system is presented in Appendix 1. In this section, the basis and the procedure for obtaining these thermodynamic parameters by using the CALPHAD method will be shortly discussed.

#### 2.3.1 Pure elements

For a pure element *i*, the Gibbs energy of the surface is described by Eq. (10) [17]:

(10) $${\text{G}}_{i}^{Surface}=\frac{2\sigma_{i}V_{i}}{r}$$

Therefore, the standard Gibbs energy of the pure element *i* including the size effect (GiNano0) will be expressed as:

(11) GiNano0=GiBulk0+2σiVir

where $V_{i}$ is the molar volume and $\sigma_{i}$ is the surface tension of the pure element. The surface tension of solid elements $\sigma_{i}^{S}$ was calculated by the approximations $\sigma_{i}^{S}\left(T_{m}\right)=1.25\sigma_{i}^{l}\left(T_{m}\right)$ (where $\sigma_{i}^{l}$ is the surface tension of the liquid element and *T_m_* is the melting point) and $\frac{d\sigma_{i}^{S}}{dT}=\frac{d\sigma_{i}^{l}}{dT}$ [18,25]. Thermodynamic and physical property data used in the present calculations are listed in Table 1.

#### 2.3.2 Binary alloys

For binary metallic alloys, the molar volume can be assumed to be additive:

(12) $$V=x_{i}V_{i}+x_{j}V_{j}$$

where *V_i_* and *V_j_* are the molar volumes of the constituents *i* and *j*. On the other hand, it is known that the surface tension of binary alloys can be calculated from Butler's equation [18] which, for an *i*–*j* binary alloy, is given as:

(13) $$\sigma =\sigma_{i}+\frac{RT}{A_{i}}\;\ln\left(\frac{x_{i}^{Surface}}{x_{i}^{Bulk}}\right)+\frac{1}{A_{i}}\left[G_{i}^{ex,Surface\;}\left(T,x_{j}^{Surface}\right)-G_{i}^{ex,Bulk\;}\left(T,x_{j}^{bulk}\right)\right]=\sigma_{j}+\frac{RT}{A_{j}}\;\ln\left(\frac{x_{j}^{Surface}}{x_{j}^{Bulk}}\right)+\frac{1}{A_{j}}\left[G_{j}^{ex,Surface\;}\left(T,x_{j}^{Surface}\right)-G_{j}^{ex,Bulk\;}\left(T,x_{j}^{bulk}\right)\right]$$

where $A_{i}$ is the molar surface area of pure *i* derived from Eq. (14) [15], $N_{0}$ is Avogadro's number, $G_{i}^{ex,Surface\;}$and $G_{i}^{ex,Bulk\;}\;$are the partial excess Gibbs energies of *i* in the surface and the bulk as a function of *T* and $x_{j}$ respectively.

(14) $$A_{i}=1.091N_{0}^{1/3}V_{i}^{2/3}$$

According to the model given in [17], it is assumed that the partial surface excess Gibbs energy is related to that of the bulk as:

(15) $$G_{i}^{ex,Surface\;}\left(T,x_{j}^{Surface}\right)=\beta^{mix}G_{i}^{ex,Bulk\;}\left(T,x_{j}^{bulk}\right)$$

where $\beta^{mix}$ stands for the ratio of the coordination number in the surface to that in the bulk. As has been demonstrated in detail by Park and Lee [17], $\beta^{mix}$ can be assumed to be 0.85 and 0.84 for liquid and solid metals, respectively. The concentration-dependent surface tension of liquid and solid binary alloys will be successively calculated from Eq. (13) and will be multiplied by the linear concentration-dependent molar volume given by Eq. (12). Consequently, the excess Gibbs energy for nano-alloys $\left(G_{i}^{ex,binary,Nano\;}\right)$ will be expressed in a Redlich–Kister type equation as a function of temperature, composition and particle radii as follows [17]:

(16) $$G_{i}^{ex,binary,Nano\;}=\sum_{i=1}^{2}\sum_{j=i+1}^{3}x_{i}x_{j}{\left(x_{i}-x_{j}\right)}^{\vartheta }L_{\vartheta ,ij}^{Nano}\left(\vartheta =0,1,2,\ldots \right)$$

where $L_{\vartheta ,ij}^{Nano}$ would be expressed as:

(17) $$L_{\vartheta ,ij}^{Nano}=f_{1}\left(\frac{1}{r}\right)+f_{2}\left(\frac{1}{r}\right)T+f_{3}\left(\frac{1}{r}\right)T.\;\ln\;T+\ldots$$

In Eq. (17), the parameters $f_{i}(1/r)$ are considered to be only functions of particle size.

#### 2.3.3 Stoichiometric intermetallic compounds

The Gibbs energy of the surface for the stoichiometric intermetallic compounds was determined individually employing the surface tension and molar volume of the corresponding chemical composition. For the SA system, Sim and Lee [15] already estimated the surface energy of the intermetallic compound Ag_3_Sn to be 0.97 N/m. Moreover, the temperature dependence of the surface tension of Ag_3_Sn was approximated to be the mean value of that of fcc and hcp Sn–Ag alloys which was $\left(-5.5897\times 10^{-5}\;\text{N}/\mathrm{mK}\right)$. The molar volume of Ag_3_Sn, derived from the volume of its unit cell at the melting point was also estimated to be $\left(4.53\times 10^{-5}\;{\text{m}}^{3}/\mathrm{mol}\right)$. In the case of Sn–Cu intermetallic compounds, which are Cu_3_Sn, Cu_6_Sn_5_, Cu_41_Sn_11_ and Cu_10_Sn_3_, Ricci et al. [32] and Lee et al. [26] previously measured the surface tension of the Cu–Sn system over the whole composition range and over a wide temperature range. Based on these two studies, the surface tension of the above-mentioned Sn–Cu intermetallic compounds has been calculated. The surface tension of solid intermetallic compound $\sigma_{IMC}^{S}$ was calculated by the approximation $\sigma_{IMC}^{S}\left(T_{m}\right)=1.25\sigma_{i}^{l}\left(T_{IMC}\right)$ at the melting point *T_m_* and $\frac{d\sigma_{IMC}^{S}}{dT}=\frac{d\sigma_{IMC}^{l}}{dT}$ [18]. The corresponding values are listed in Table 2.

Similarly, the molar volume of Sn–Cu intermetallic compounds was derived from the volume of their unit cell and their volume expansion coefficient, as given in Table 2. Consequently, the general Gibbs energy of intermetallic compounds including the size effect $\left(G_{IMC}^{Nano}\right)$ will be described as follows:

(18) $$G_{IMC}^{Nano}=G_{IMC}^{Bulk}+\frac{2\sigma_{IMC}V_{IMC}}{r}$$

#### 2.3.4 Ternary alloys

It is noteworthy to mention that the ternary solubility for most of the binary compounds in the SAC system is insignificant, and since no ternary compounds have been reported experimentally for SAC alloys, all the compounds were considered to be binary compounds with no ternary solubility. Therefore the corresponding thermodynamic descriptions were adopted from the three Sn–Ag, Sn–Cu and Ag–Cu binary systems [22]. In the case of the ternary liquid phase in the SAC nano-system, the thermodynamic parameters are taken directly from the liquid bulk.

## 3 Discussion

### 3.1 Synthesis and characterization of SAC nanoparticles

**Fig. 1:**
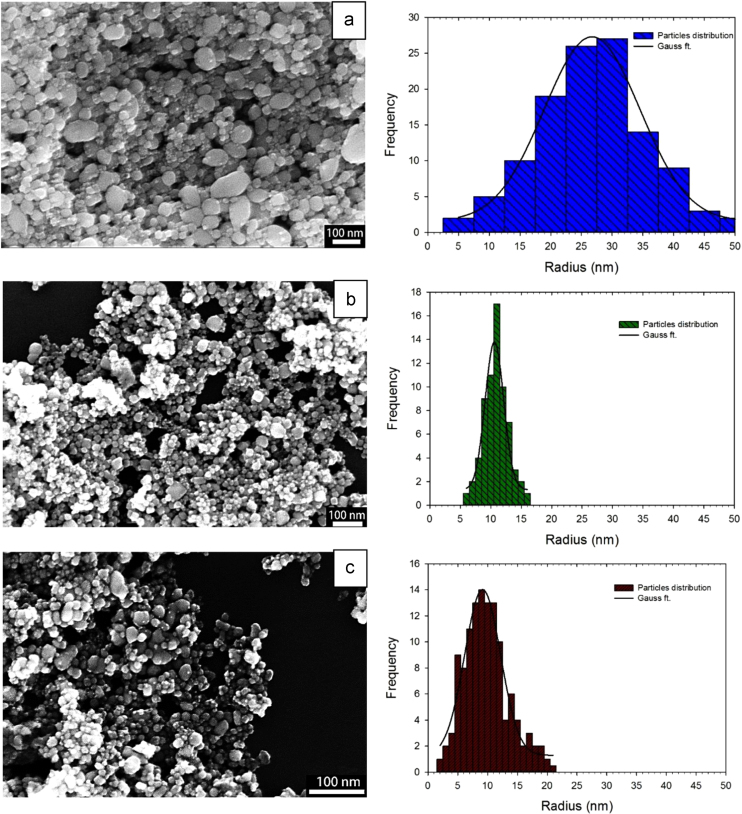
SEM images of SAC alloy nanoparticles synthesized using different molar ratios of PVP to SAC; (a) 0.05, (b) 0.35 and (c) 0.4. The corresponding particle size and size distribution is presented as a histogram on the right side of each image.

**Fig. 2:**
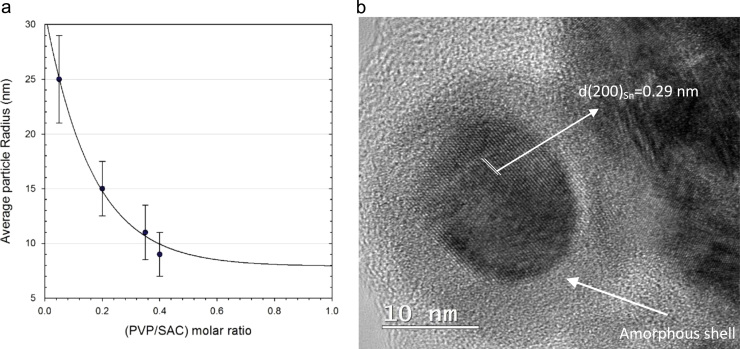
The effect of PVP concentration on average particle size (*r*) of SAC nanoparticles (a) and a typical HRTEM image of a nanoparticle consisting of a well-crystallized β-Sn core covered with an amorphous layer (b).

Fig. 1 exhibits the SEM images of SAC alloy nanoparticles synthesized using different molar ratios of PVP to SAC; (a) 0.05 (b) 0.35 and (c) 0.4. The corresponding particle size and size distribution is also presented as a histogram on the right side of each image, indicating the average particle radius of 25, 11 and 9 nm, respectively. As inferred from these figures, nearly spherical nanoparticles with a relatively narrow size distribution have been successfully produced by employing PVP as surfactant. Without protection by the voluminous PVP molecules, the as-reduced nanocrystals will start to agglomerate rather quickly. However, the presence of PVP provides excellent protection and prevents any significant sintering of the growing nanoparticles, thus controlling the size of the nanoparticles [35–37]. Fig. 2(a) shows a plot of the average particle size of the nanoparticles as a function of PVP concentration. It was found that by introducing PVP to the precursors, the particle size decreased drastically, while a further increase in the ratio of PVP to SAC beyond 0.4 altered the particle size only slightly, which is probably due to the fact that the surface of the particles becomes saturated by the surfactant molecules.

**Fig. 3:**
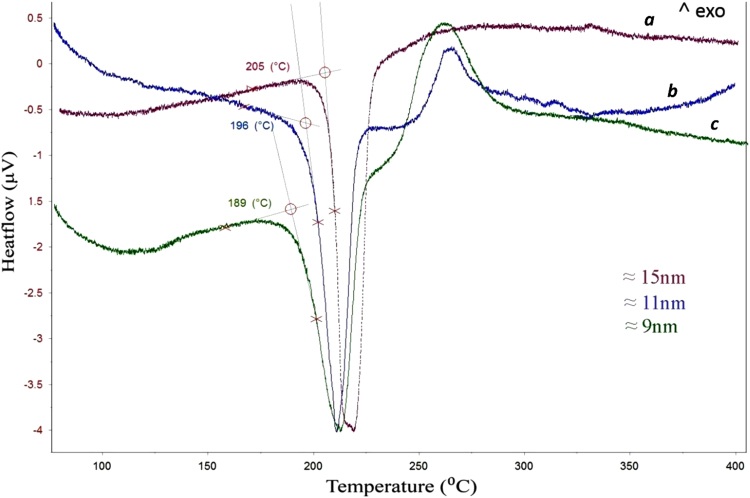
DSC curves of SAC nanoparticles with the average particle radius of 15, 11 and 9 nm as marked by a, b and c, respectively. The melting onset temperature is highlighted as a circle on each curve.

Fig. 3 depicts the DSC curves of SAC nanoparticles with the average particle radius of 9, 11 and 15 nm. As marked by circles in this figure, the extrapolated onsets were determined to be 189, 196 and 205 °C, respectively. It should be pointed out that the onset of the DSC peak was evaluated as the eutectic point (invariant transformation) of the SAC alloy with a given average particle size, determined from SEM/TEM images. As can be seen for all three samples, the deviation of the DSC curve from the base line starts already at a considerably lower temperature. It must be assumed that this indicates the eutectic temperature for the smallest particles in each sample whereas the extrapolated onset refers to the melting of the majority of the particles with the given average particle size. This is indicated by a corresponding error bar in the figure. As inferred from this figure, a melting point depression of nearly 30 °C was achieved for the nanoparticles with a radius of 9 nm which is already quite close to the eutectic temperature of the conventional Sn–Pb solder alloys.

Apart from the eutectic effect, one can clearly distinguish in this figure exothermic peaks between 250 and 300 °C in the DSC curves of the samples with smaller particle sizes (9 and 11 nm). A detailed explanation of this phenomenon falls outside the scope of this article and has been reported by Roshanghias et al. [20]. Nevertheless, a short explanation is given. As shown in the high-resolution TEM image in Fig. 2(b), it can be implied that the surface of the nanoparticles appears to be covered by an amorphous layer, enriched with Sn and O. The observed exothermic peak was associated with the crystallization of this amorphous SnO shell, according to the following possible chemical reaction:

(19) $${\mathrm{SnO}}_{\left(\text{amorphous}\right)}\to {\mathrm{SnO}}_{\left(\text{crystalline}\right)}+\mathrm{\Delta Q}↑$$

Therefore, as the particle size decreases and the surface to volume ratio increases, the exothermic peak associated with the crystallization of this superficial amorphous shell becomes more and more evident. This amorphous surface layer may represent a major drawback for the practical use of tin-based nanoparticles, prepared by chemical reduction, as reliable solders. A possible solution could be the use of an acidic soldering flux which is able to dissolve this layer [2–8].

### 3.2 Phase diagram assessment

**Fig. 4:**
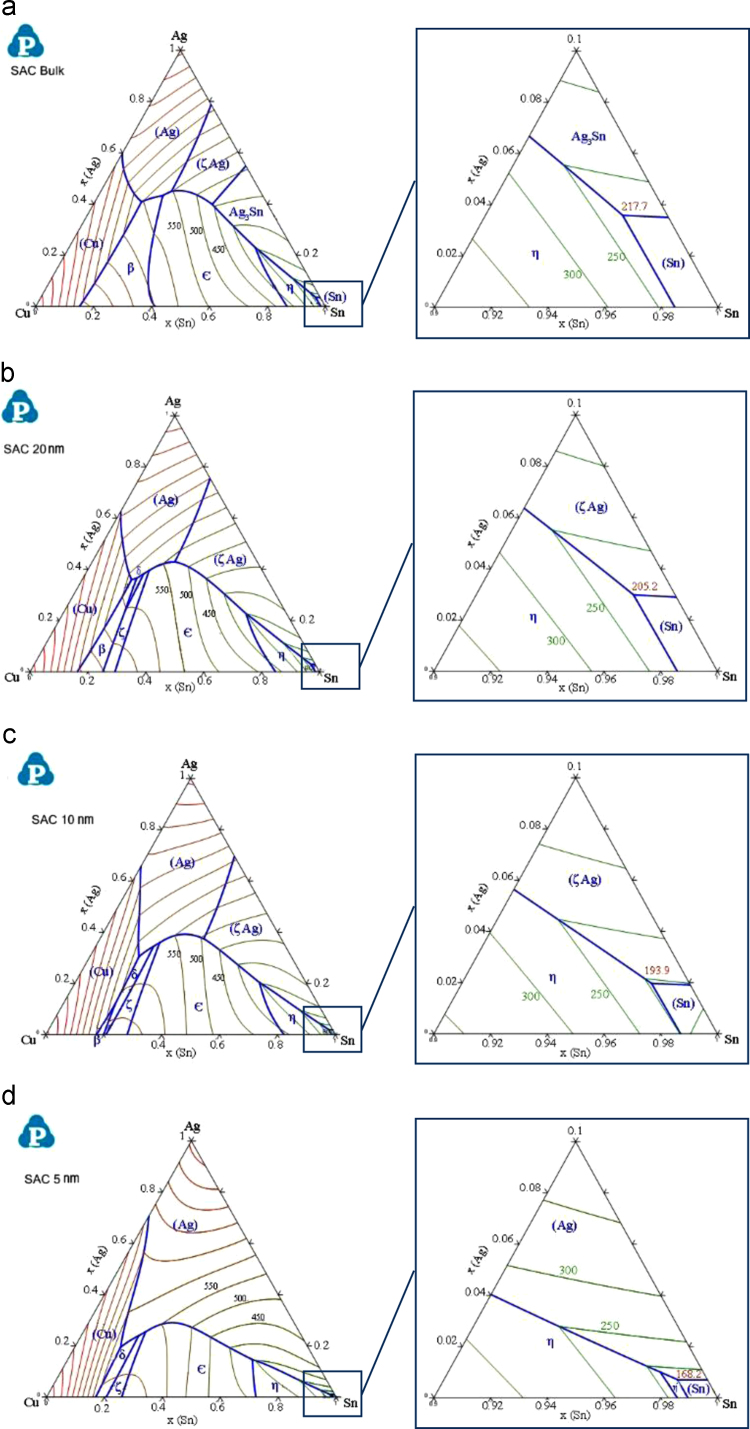
The calculated liquidus projection of the Sn–Ag–Cu ternary system of different particle radius, bulk (a), 20 nm (b), 10 nm (c) and 5 nm (d).

In Fig. 4, the calculated liquidus projection of the SAC ternary phase diagram has been plotted for three different radii of nanoparticles (5, 10, 20 nm) together with the bulk SAC diagram. The Sn-rich corner has been magnified in the figures demonstrating the variation of the eutectic point in temperature and composition, as well.

**Fig. 5:**
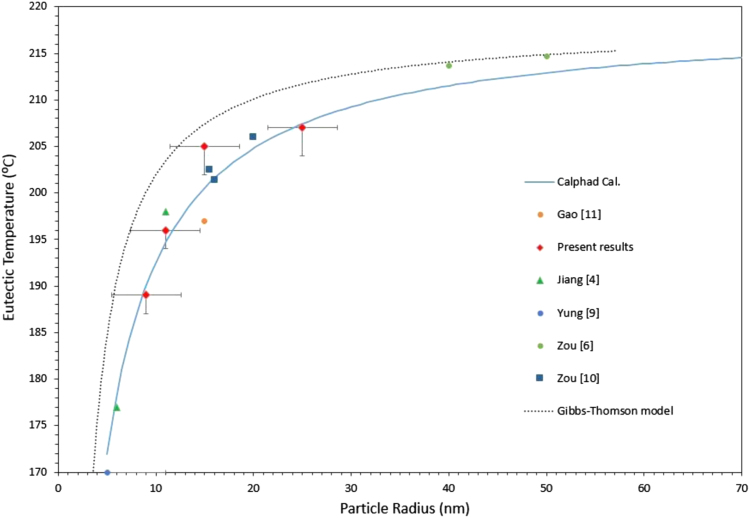
Eutectic temperature dependence of SAC alloys on particle size, predicted by the CALPHAD model (full line) and the Gibbs–Thomson model (dotted line) compared with experimental results (symbols).

As inferred from Fig. 4, decreasing the size of the particles not only depresses the transformation temperatures in the system, it also affects the course of the monovariant lines in the liquidus projection, and thus the eutectic points. The eutectic temperature obtained from the CALPHAD modeling has been plotted as a function of the particle radius in Fig. 5. The figure shows clearly that the calculated eutectic temperatures are consistent with the published experimental data and the results obtained from the DSC measurements in the present study.

The prominent size dependent melting point depression behavior of materials in the nano-scale can be clearly inferred from this curve. Furthermore, it can be seen that the CALPHAD model shows even better agreement with the experiments than the curve calculated with Eq. (1) according to the Gibbs–Thomson model which is also included in Fig. 5. The eutectic temperature *T_eut_*, obtained from the CALPHAD model, can be expressed as a function of the particle radius (*r*) by a nonlinear hyperbolic regression with a deviation of less than 2 °C:

(20) $$T_{eut}\left(\pm 2\;{}^{\circ}\text{C}\right)=\frac{218.7r}{1.362+r}$$

**Fig. 6:**
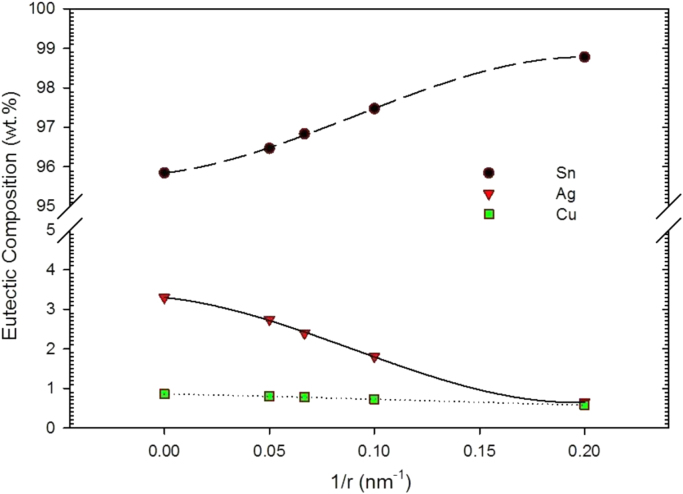
The variation in the eutectic composition of SAC alloys with the particle radius (*r*).

The variation of the eutectic composition with the reciprocal particle radius is shown in Fig. 6. As one can observe, the eutectic composition moves closer to the Sn-rich corner as the particle size reduces. Whereas the Cu concentration reduces slightly, the Sn content increases markedly with decreasing particle size which leads at the same time to a decrease of the Ag content. For a particle radius of 10 nm the eutectic point is found at a temperature of 193.9 °C and a composition of Sn-1.8 wt% Ag-0.7 wt% Cu. This shift toward the Sn-rich corner of the phase diagram is roughly consistent with the model proposed by Lee et al. [38] for binary alloys, in which the eutectic composition of nano-alloys moved to the side of the component with the lower melting point.

Nonetheless, it is important to point out that the present assessment was mainly addressed to the Sn-rich corner of the SAC system, since the experimental data available in the literature of SAC nanoparticles as well as the experimental results in the present study are mostly referring to this part of the ternary system [6–11]. Further experimental work is required to improve the thermodynamic description of the SAC nano-system in other regions of the ternary phase diagram.

## 4 Conclusion

On the basis of the CALPHAD method, the phase equilibria in the ternary Sn–Ag–Cu (SAC) system have been reassessed including the size effect for nanomaterials. In order to validate the reliability of the thermodynamic model, near eutectic Sn–3.8Ag–0.7Cu nanoparticles of different size were synthesized via a chemical reduction technique by employing PVP as surfactant agent and characterized by electron microscopy and DSC investigations. It is shown that the eutectic point predicted by the model for SAC nanoparticles is in good agreement with the experimental data. The size dependent melting point depression behavior of SAC alloys was calculated by the model and proved by the DSC measurements. Consequently, based on the proposed model, the eutectic temperature ($T_{eut})$ of the SAC nanoparticles with the radius *r* can be calculated as: $T_{eut}\left(\pm 2\;{}^{\circ}\text{C}\right)=\frac{218.7r}{1.362+r}\;$which can provide a handy means for verification in future experimental studies. The calculations also resulted in a change of the eutectic composition towards the Sn-rich corner with decreasing particle size.

## Figures and Tables

**Table 1:** Thermodynamic and physical properties of Sn, Ag and Cu.

Properties	Equations (*T* in K)	References
Surface tension (N/m)	$\sigma_{Ag}^{L}=1.207-2.28\times 10^{-4}T$	[26]
	$\sigma_{Ag}^{S}=1.675-0.47\times 10^{-3}T$	[27]
	$\sigma_{Sn}^{L}=0.5828-0.8343\times 10^{-4}T$	[28]
	$\sigma_{Sn}^{S}=0.729-1.4\times 10^{-4}T$	[28]
	$\sigma_{Cu}^{L}=1.624-0.226\times 10^{-3}T$	[29]
	$\sigma_{Cu}^{S}=1.9535-0.226\times 10^{-3}T$	[18]
Molar volume $\left({\text{m}}^{3}/\mathrm{mol}\right)$	$V_{Ag}^{L}=1.0198\times 10^{-5}-1.1368\times 10^{-9}T$	[30]
	$V_{Ag}^{S}=1.12066\times 10^{-5}$	[30]
	$V_{Sn}^{L}=1.70\times 10^{-5}$	[31]
	$V_{Sn}^{S}=1.62\times 10^{-5}$	[31]
	$V_{Cu}^{L}=6.95\times 10^{-6}-8.08\times 10^{-10}T$	[29]
	$V_{Cu}^{S}=7.09\times 10^{-6}$	[18]

**Table 2:** Thermodynamic and physical properties of the Sn–Ag–Cu stoichiometric intermetallic compounds.

Properties	Equations (*T* in K)	References
Surface tension(N/m)	$\sigma_{Cu6Sn5}^{S}=0.6460+4.90\times 10^{-5}T$	[26,32]
	$\sigma_{Cu3Sn}^{S}=0.7597+7.356\times 10^{-5}T$	[26,32]
	$\sigma_{Cu41Sn11}^{S}=0.854+5.376\times 10^{-5}T$	[26,32]
	$\sigma_{Cu10Sn3}^{S}=0.802+6.51\times 10^{-5}T$	[26,32]
	$\sigma_{Ag3Sn}^{S}=0.97-5.5897\times 10^{-5}T$	[15]
Molar volume $\left({\text{m}}^{3}/\mathrm{mol}\right)$	$V_{Cu6Sn5}^{S}=1.2\times 10^{-5}+6.36\times 10^{-10}T$	[33,34]
	$V_{Cu3Sn}^{S}=9.7\times 10^{-6}+5.23\times 10^{-10}T$	[33,34]
	$V_{Cu41Sn11}^{S}=9.3\times 10^{-6}+5.01\times 10^{-10}T$	[33,34]
	$V_{Cu10Sn3}^{S}=9.5\times 10^{-6}+5.12\times 10^{-10}T$	[33,34]
	$V_{Ag3Sn}^{S}=4.53\times 10^{-5}$	[15]
