# Supplementary material for: Sn–Ag–Cu nanosolders: Melting behavior and phase diagram prediction in the Sn-rich corner of the ternary system
Source: CALPHAD. 2015 Jun;49:101–9. doi: 10.1016/j.calphad.2015.04.003 (PMC4456117; doi:10.1016/j.calphad.2015.04.003)
Supplement: Supplementary file 1 — Supplementary material [file mmc1.doc]

$======================================================================

Appendix 1

The general Gibbs free energy description of Sn-Ag-Cu system, including the size effect.

The Function RR in this database corresponds to reciprocal radius (1/r) in nanometer.

$======================================================================

ELEMENT /- ELECTRON_GAS 0. 0. 0. !

ELEMENT VA VACUUM 0. 0. 0. !

ELEMENT AG FCC_A1 1.0787E+02 5.7446E+03 4.2551E+01 !

ELEMENT CU FCC_A1 6.3546E+01 5.0041E+03 3.3150E+01 !

ELEMENT SN BCT_A5 1.1871E+02 6.3220E+03 5.1195E+01 !

FUNCTION RR 298.15 0.0E+08; 3000 N Reciprocal_Radius_[nm] !

FUNCTION UN_ASS 298.15 0; 300 N !

FUNCTION GHSERAG 2.98140E+02 -7209.512+118.202013*T-23.8463314*T*LN(T)

-.001790585*T**2-3.98587E-07*T**3-12011*T**(-1); 1.23493E+03 Y

-15095.252+190.266404*T-33.472*T*LN(T)+1.411773E+29*T**(-9);

3.00000E+03 N !

FUNCTION GHSERCU 298.15

-7770.458+130.485235*T-24.112392*T*LN(T)-2.65684E-3*T**2+0.129223E-6*T**3

+52478*T**(-1); 1357.77 Y

-13542.026+183.803828*T-31.38*T*LN(T)+364.167E27*T**(-9); 3200 N !

FUNCTION GHSERSN 100.00

-7958.517+122.765451*T-25.858*T*LN(T)+0.51185E-3*T**2-3.192767E-6*T**3

+18440*T**(-1); 250 Y

-5855.135+65.443315*T-15.961*T*LN(T)-18.8702E-3*T**2+3.121167E-6*T**3

-61960*T**(-1); 505.08 Y

2524.724+4.005269*T-8.2590486*T*LN(T)-16.814429E-3*T**2+2.623131E-6*T**3

-1081244*T**(-1)-123.07E23*T**(-9); 800 Y

-8256.959+138.99688*T-28.4512*T*LN(T)-123.07E23*T**(-9); 3000 N !

TYPE_DEFINITION % SEQ * !

DEFINE_SYSTEM_DEFAULT SPECIE 2 !

DEFAULT_COMMAND DEF_SYS_ELEM VA !

PHASE LIQUID % 1 1 !

CONSTITUENT LIQUID :AG,CU,SN : !

PARAMETER G(LIQUID,AG;0) 298.15

3815.564+109.310993*T-23.8463314*T*LN(T)

-1.790585E-3*T**2-0.398587E-6*T**3

-12011*T**(-1)-1044.905E-23*T**7

+2.462e-5*RR-1.906e-9*RR*T-5.18e-13*RR*T**2; 1234.93 Y

-3587.111+180.964656*T-33.472*T*LN(T)

+2.462e-5*RR-1.906e-9*RR*T-5.18e-13*RR*T**2; 3000 N !

PARAMETER G(LIQUID,CU;0) 298.15

5194.277+2.258E-5*RR+120.973331*T-5.1669E-10*RR*T

-24.112392*T*LN(T)

-2.65684E-3*T**2-3.6522E-13*RR*T**2+0.129223E-6*T**3

+52478*T**(-1)-584.89E-23*T**7; 1357.77 Y

-46.54+2.258E-5*RR+173.881484*T-5.1669E-10*RR*T-31.38*T*LN(T)

-3.6522E-13*RR*T**2; 3200 N !

PARAMETER G(LIQUID,SN;0) 100

-855.425+108.677684*T-25.858*T*LN(T)+0.51185E-3*T**2-3.192767E-6*T**3

+18440*T**(-1)+147.031E-20*T**7

+1.9815E-05*RR-2.8366E-09*T*RR; 250 Y

1247.957+51.355548*T-15.961*T*LN(T)-18.8702E-3*T**2+3.121167E-6*T**3

-61960*T**(-1)+147.031E-20*T**7

+1.9815E-05*RR-2.8366E-09*T*RR; 505.08 Y

9496.31-9.809114*T-8.2590486*T*LN(T)-16.814429E-3*T**2+2.623131E-6*T**3

-1081244*T**(-1)

+1.9815E-05*RR-2.8366E-09*T*RR; 800 Y

-1285.372+125.182498*T-28.4512*T*LN(T)

+1.9815E-05*RR-2.8366E-09*T*RR; 3000 N !

PARAMETER G(LIQUID,AG,SN;0) 298.15 -4908.719-4.702*T

-2.943E-05*RR+1.794E-08*RR*T; 6000 N Sim-Lee !

PARAMETER G(LIQUID,AG,SN;1) 298.15 -16988+4.702*T

-1.857E-05*RR+1.501E-09*RR*T; 6000 N Sim-Lee !

PARAMETER G(LIQUID,AG,SN;2) 298.15 -6840.2

-4.942E-07*RR+3.157E-09*RR*T; 6000 N Sim-Lee !

PARAMETER G(LIQUID,AG,CU;0) 298.15 17384.37-10.89e-6*RR-4.46438*T

+4.25e-9*RR*T; 6000 N !

PARAMETER G(LIQUID,AG,CU;1) 298.15 1660.74-13.65e-6*RR-2.31516*T

+7.35e-9*RR*T; 6000 N !

PARAMETER G(LIQUID,AG,CU;2) 298.15 -6.67e-6*RR

+2.70e-9*RR*T; 6000 N !

PARAMETER G(LIQUID,CU,SN;0) 298.15 -9002.8-5.8381*T

-5.1155E-05*RR+0.1373E-07*RR*T; 3000 N !

PARAMETER G(LIQUID,CU,SN;1) 298.15 -18936.316+2.339*T

-4.2310E-05*RR+0.1687E-07*RR*T; 3000 N !

PARAMETER G(LIQUID,CU,SN;2) 298.15 -14122.6+52.942*T-7.057*T*LN(T)

-8.4525E-06*RR+0.1085E-08*RR*T; 3000 N !

$PARAMETER G(LIQUID,AG,CU,SN;0) 298.15 -48000; 3000 N !

PARAMETER G(LIQUID,AG,CU,SN;0) 298.15 -43626.3134-29.6939949*T; 3000 N !

PARAMETER G(LIQUID,AG,CU,SN;1) 298.15 -4264.2128-72.6592364*T; 3000 N !

PARAMETER G(LIQUID,AG,CU,SN;2) 298.15 -44369.0813+19.7670486*T; 3000 N !

PHASE BCT_A5 % 1 1 !

CONSTITUENT BCT_A5 :AG,SN% : !

PARAMETER G(BCT_A5,AG;0) 298.15

-3025.412+118.202013*T-23.8463314*T*LN(T)-1.790585E-3*T**2-0.398587E-6*T**3

-12011*T**(-1)

+3.942e-5*RR-1.1061e-8*RR*T; 1234.93 Y

-10911.152+190.266404*T-33.472*T*LN(T)+1411.773E26*T**(-9)

+3.942e-5*RR-1.1061e-8*RR*T; 3000 N !

PARAMETER G(BCT_A5,SN;0) 100.00

-7958.517+122.765451*T-25.858*T*LN(T)+0.51185E-3*T**2-3.192767E-6*T**3

+18440*T**(-1)

+2.48E-05*RR-4.7628E-09*T*RR; 250 Y

-5855.135+65.443315*T-15.961*T*LN(T)-18.8702E-3*T**2+3.121167E-6*T**3

-61960*T**(-1)

+2.48E-05*RR-4.7628E-09*T*RR; 505.08 Y

2524.724+4.005269*T-8.2590486*T*LN(T)-16.814429E-3*T**2+2.623131E-6*T**3

-1081244*T**(-1)-123.07E23*T**(-9)

+2.48E-05*RR-4.7628E-09*T*RR; 800 Y

-8256.959+138.99688*T-28.4512*T*LN(T)-123.07E23*T**(-9)

+2.48E-05*RR-4.7628E-09*T*RR; 3000 N !

PARAMETER G(BCT_A5,AG,SN;0) 298.15 18358.8; 6000 N !

PHASE FCC_A1 % 2 1 1 !

CONSTITUENT FCC_A1 :AG%,CU%,SN : VA : !

PARAMETER G(FCC_A1,AG:VA;0) 298.15

-7209.512+118.202013*T-23.8463314*T*LN(T)

-1.790585E-3*T**2-0.398587E-6*T**3-12011*T**(-1)

+3.224e-5*RR-5.1104e-9*RR*T; 1234.93 Y

-15095.252+190.266404*T-33.472*T*LN(T)

+1411.773E26*T**(-9)

+3.224e-5*RR-5.1104e-9*RR*T; 3000 N !

PARAMETER G(FCC_A1,CU:VA;0) 298.15

-7770.458+2.77E-5*RR-3.2047E-9*RR*T+130.485235*T-24.112392*T*LN(T)

-2.65684E-3*T**2+0.129223E-6*T**3

+52478*T**(-1); 1357.77 Y

-13542.02+2.77E-5*RR-3.2047E-9*RR*T+183.803828*T-31.38*T*LN(T)

+364.167E27*T**(-9); 3200 N !

PARAMETER G(FCC_A1,SN:VA;0) 298.15

-345.135+56.983315*T-15.961*T*LN(T)-18.8702E-3*T**2+3.121167E-6*T**3

-61960*T**(-1)

+2.48E-05*RR-4.7628E-09*T*RR; 505.08 Y

8034.724-4.454731*T-8.2590486*T*LN(T)-16.814429E-3*T**2+2.623131E-6*T**3

-1081244*T**(-1)-123.07E23*T**(-9)

+2.48E-05*RR-4.7628E-09*T*RR; 800 Y

-2746.959+130.53688*T-28.4512*T*LN(T)-123.07E23*T**(-9)

+2.48E-05*RR-4.7628E-09*T*RR; 3000 N !

PARAMETER G(FCC_A1,AG,SN:VA;0) 298.15 4381.8+12.57*T

-1.126E-04*RR+5.149E-08*RR*T; 6000 N !

PARAMETER G(FCC_A1,AG,SN:VA;1) 298.15 -41594.5

-1.319E-4*RR+8.191E-08*RR*T; 6000 N !

PARAMETER G(FCC_A1,AG,CU:VA;0) 298.15 36772.58-11.02847*T; 6000 N !

PARAMETER G(FCC_A1,AG,CU:VA;1) 298.15 -4612.43 +0.28869*T; 6000 N !

PARAMETER G(FCC_A1,CU,SN:VA;0) 298.15 -11106.95+2.0791*T; 3000 N !

PARAMETER G(FCC_A1,CU,SN:VA;1) 298.15 -15718.018+5.9254696*T; 3000 N !

PHASE BCC_A2 % 2 1 3 !

CONSTITUENT BCC_A2 :AG,CU,SN : VA : !

PARAMETER G(BCC_A2,AG:VA;0) 298.15

-3809.512+117.152013*T-23.8463314*T*LN(T)-1.790585E-3*T**2-0.398587E-6*T**3

-12011*T**(-1)+3.224e-5*RR-5.1104e-9*RR*T; 1234.93 Y

-11695.252+189.216404*T-33.472*T*LN(T)+1411.773E26*T**(-9)

+3.224e-5*RR-5.1104e-9*RR*T; 3000 N !

PARAMETER G(BCC_A2,CU:VA;0) 298.15

-3753.458+129.230235*T-24.112392*T*LN(T)-2.65684E-3*T**2+0.129223E-6*T**3

+52478*T**(-1)+2.77E-5*RR-3.2047E-9*RR*T; 1357.77 Y

-9525.026+182.548828*T-31.38*T*LN(T)+364.167E27*T**(-9)

+2.77E-5*RR-3.2047E-9*RR*T; 3200 N !

PARAMETER G(BCC_A2,SN:VA;0) 100

-3558.517+116.765451*T-25.858*T*LN(T)+0.51185E-3*T**2-3.192767E-6*T**3

+18440*T**(-1)+2.48E-05*RR-4.7628E-09*T*RR; 250 Y

-1455.135+59.443315*T-15.961*T*LN(T)-18.8702E-3*T**2+3.121167E-6*T**3

-61960*T**(-1)+2.48E-05*RR-4.7628E-09*T*RR; 505.08 Y

6924.724-1.994731*T-8.2590486*T*LN(T)-16.814429E-3*T**2+2.623131E-6*T**3

-1081244*T**(-1)-123.07E23*T**(-9)+2.48E-05*RR-4.7628E-09*T*RR; 800 Y

-3856.959+132.99688*T-28.4512*T*LN(T)-123.07E23*T**(-9)

+2.48E-05*RR-4.7628E-09*T*RR; 3000 N !

PARAMETER G(BCC_A2,AG,CU:VA;0) 298.15 36772.58-11.02847*T; 6000 N !

PARAMETER G(BCC_A2,AG,CU:VA;1) 298.15 -4612.43 +0.28869*T; 6000 N !

PARAMETER G(BCC_A2,AG,SN:VA;0) 298.15 7000; 6000 N !

PARAMETER G(BCC_A2,CU,SN:VA;0) 298.15 -32656.8+25.015776*T; 3000 N !

PARAMETER G(BCC_A2,CU,SN:VA;1) 298.15 -13862.5-32.0218*T; 3000 N !

PARAMETER G(BCC_A2,CU,SN:VA;2) 298.15 -4175.47+5.0083*T; 3000 N !

PARAMETER G(BCC_A2,AG,CU,SN:VA;0) 298.15 -95941.72; 3000 N !

PHASE HCP_A3 % 2 1 .5 !

CONSTITUENT HCP_A3 :AG,CU,SN : VA : !

PARAMETER G(HCP_A3,AG:VA;0) 298.15

-6909.512+118.502013*T-23.8463314*T*LN(T)-1.790585E-3*T**2-0.398587E-6*T**3

-12011*T**(-1)

+3.802e-5*RR-1.1061e-8*RR*T; 1234.93 Y

-14795.252+190.566404*T-33.472*T*LN(T)+1411.773E26*T**(-9)

+3.802e-5*RR-1.1061e-8*RR*T; 3000 N !

PARAMETER G(HCP_A3,CU:VA;0) 298.15

-7170.458+130.685235*T-24.112392*T*LN(T)-2.65684E-3*T**2+0.129223E-6*T**3

+52478*T**(-1)

+3.802e-5*RR-1.1061e-8*RR*T; 1357.77 Y

-12942.026+184.003828*T-31.38*T*LN(T)+364.167E27*T**(-9)

+3.802e-5*RR-1.1061e-8*RR*T; 3200.00 N !

PARAMETER G(HCP_A3,SN:VA;0) 298.15

-1955.135+57.797315*T-15.961*T*LN(T)-18.8702E-3*T**2+3.121167E-6*T**3

-61960*T**(-1)

+2.45156E-05*RR-3.2947E-09*T*RR; 505.08 Y

6424.724-3.640731*T-8.2590486*T*LN(T)-16.814429E-3*T**2+2.623131E-6*T**3

-1081244*T**(-1)-123.07E23*T**(-9)

+2.45156E-05*RR-3.2947E-09*T*RR; 800 Y

-4356.959+131.35088*T-28.4512*T*LN(T)-123.07E23*T**(-9)

+2.45156E-05*RR-3.2947E-09*T*RR; 3000 N !

PARAMETER G(HCP_A3,AG,SN:VA;0) 298.15 297.67+10.62*T

-1.058E-04*RR+5.304E-08*RR*T; 6000 N !

PARAMETER G(HCP_A3,AG,SN:VA;1) 298.15 -38953.88

-1.124E-04*RR+8.137E-08*RR*T; 6000 N !

PARAMETER G(HCP_A3,AG,CU:VA;0) 298.15 +36772.58-11.02847*T; 6000 N !

PARAMETER G(HCP_A3,AG,CU:VA;1) 298.15 -4612.43+0.28869*T; 6000 N !

PARAMETER G(HCP_A3,CU,SN:VA;0) 298.15 5000; 1000 N !

PHASE AGSB_ORTHO % 2 .75 .25 !

CONSTITUENT AGSB_ORTHO :AG: AG,SN : !

PARAMETER G(AGSB_ORTHO,AG:AG;0) 2.98140E+02 +GHSERAG#+5000+3.940E-05*RR

-1.10609E-08*RR*T; 3.00000E+03 N REF:0 !

PARAMETER G(AGSB_ORTHO,AG:SN;0) 2.98150E+02 -11085.3+3.495E-05*RR

+110.01471*T-6.49E-09*RR*T

-23.18*T*LN(T)-.00359*T**2+4389.5*T**(-1); 6.00000E+03 N REF:0 !

PARAMETER G(AGSB_ORTHO,AG:AG,SN;0) 2.98140E+02 0.0 ; 3.00000E+03 N

REF:0 !

PHASE DIAMOND_A4 % 1 1.0 !

CONSTITUENT DIAMOND_A4 :SN : !

PARAMETER G(DIAMOND_A4,SN;0) 1.00000E+02 -9579.608+114.007785*T

-22.972*T*LN(T)-.00813975*T**2+2.7288E-06*T**3+25615*T**(-1)

+2.48E-05*RR-4.7628E-09*T*RR; 2.98140E+02

Y

-9063.001+104.84654*T-21.5750771*T*LN(T)-.008575282*T**2

+1.784447E-06*T**3-2544*T**(-1)

+2.48E-05*RR-4.7628E-09*T*RR; 8.00000E+02 Y

-10909.351+147.396535*T-28.4512*T*LN(T)

+2.48E-05*RR-4.7628E-09*T*RR; 3.00000E+03 N REF:0 !

PHASE CU6SN5_P % 2 .545 .455 !

CONSTITUENT CU6SN5_P :CU :SN : !

PARAMETER G(CU6SN5_P,CU:SN;0) 298.15 -7129.7+0.4059*T+0.545*GHSERCU

+0.455*GHSERSN

+1.5247E-05*RR+1.9799E-09*RR*T+6.2434E-14*RR*T**2; 3000 N !

PHASE CU3SN % 2 .75 .25 !

CONSTITUENT CU3SN :CU : SN : !

PARAMETER G(CU3SN,CU:SN;0) 298.15 -8194.2-0.2043*T+0.75*GHSERCU

+0.25*GHSERSN

+1.4738E-05*RR+2.22E-09*RR*T+7.698E-14*RR*T**2; 3000 N !

PHASE CUIN_ETA % 3 .545 .122 .333 !

CONSTITUENT CUIN_ETA :CU :CU, SN : SN : !

PARAMETER G(CUIN_ETA,CU:CU:SN;0) 298.15 3200+2*T

+.667*GHSERCU+.333*GHSERSN

+1.5247E-05*RR+1.9799E-09*RR*T+6.2434E-14*RR*T**2; 6000 N !

PARAMETER G(CUIN_ETA,CU:SN:SN;0) 298.15 -6869.5-0.1589*T

+.545*GHSERCU+.455*GHSERSN

+1.5247E-05*RR+1.9799E-09*RR*T+6.2434E-14*RR*T**2; 6000 N !

PARAMETER G(CUIN_ETA,CU:CU,SN:SN;0) 298.15 -8300; 6000 N !

PHASE CU41SN11 % 2 .788 .212 !

CONSTITUENT CU41SN11 :CU : SN : !

PARAMETER G(CU41SN11,CU:SN;0) 298.15 -6323.5-1.2808*T+0.788*GHSERCU

+0.212*GHSERSN

+1.5880E-05*RR+1.857E-09*RR*T+5.39E-14*RR*T**2; 3000 N !

PHASE CU10SN3 % 2 .769 .231 !

CONSTITUENT CU10SN3 :CU :SN : !

PARAMETER G(CU10SN3,CU:SN;0) 298.15 -6655.1-1.485*T+0.769*GHSERCU

+0.231*GHSERSN

+1.520E-05*RR+2.059E-09*RR*T+6.67E-14*RR*T**2; 3000 N !
